# Recent Improvements in Adult Wilms Tumor Diagnosis and Management: Review of Literature

**DOI:** 10.15586/jkcvhl.v10i3.281

**Published:** 2023-08-11

**Authors:** Vishnupriya Sakthivel, Ismail Z Adeeb, Devashree Vijayabalan

**Affiliations:** Department of Pharmacy Practice, PSG College of Pharmacy, Coimbatore, India

**Keywords:** adults, chemotherapy, nephroblastoma, radiotherapy, WT gene

## Abstract

Wilms tumor, also denoted as nephroblastoma, an embryonal type of renal cancer, is the most common cancer that affects children in the first 5 years of life. Wilms tumor is very rarely seen in adults. Both adults and children showcase varied clinical symptoms. The metastasis of tumor in both adults and children are not uncommon. Though histological differences between children and adults are insignificant, the prognosis of adult Wilms tumors compared to children is abysmal. Despite remarkable advancements in oncology, no standard treatment protocol exists for Wilms tumor in adults. Children Wilms tumor treatment protocol is currently followed for adults with some changes. In this article, we reviewed the available treatment options for Wilms tumor in adults and protocols followed widely.

## Introduction

Wilms tumor (WT), also denoted as nephroblastoma, is a type of cancer that affects the kidneys. It is most observed in children in the first 5 years of life, with the highest incidence between 3 and 4 years of age (1). US National Wilms Tumor Study reported that 38 months is the median onset age for tumors in children. There is no distinguished report based on the gender difference. But in a few Asian countries, females are more likely to have WT than males (M: F=1:4) (2).

Wilms tumor is widely seen in Africans and African Americans. On the other hand, it is the least common type of cancer in the East Asian population. Asian patients also had tumors with lesser unfavorable histology and were likely to develop lower-stage disease and experience better clinical results. The same rate is seen in Europeans and North Americans.

Wilms tumor is sparsely seen in the adult population and accounts for less than 1% of all kidney cancers with an incidence of 0.2 per million per year (1). An adult Wilms tumor is often diagnosed mistakenly as renal cell carcinoma. This becomes evident after the removal of the kidney or nephrectomy (3).

Adults and children with Wilms tumor are presented with different symptoms. Adults usually experience abdominal pain and hematuria, while children’s tumors are painless and often cause an easily palpable enlarged abdomen (4). Histological and radiological differences between adult and child Wilms tumors are not significantly seen. Distant metastases to the lung and liver are common in both adults and children. Tumors rarely metastasize to bone, skin, bladder, colon, central nervous system, and contralateral kidney (1).

Compared with children, the clinical outcome in adults has been poor due to various factors, as no specific regimen or protocol is available for treating Wilms tumor in adults. To improve feasibility and survival, the application or adjustment of pediatric protocols, which were formulated by The Children’s Oncology Group (COG) and the International Society of Pediatric Oncology (SIOP) to adults, have been suggested considering recent experiences (3). Despite these reports, due to the limited availability of the information, surgeons and medical oncologists find it challenging to treat adult Wilms tumor cases. For patients over 16, the preferred treatment is primary radical nephrectomy with subsequent chemotherapy (4). Yet, there is a subsequent gap between diagnosing and initiating pertinent situation-based treatment for adult Wilms tumors.

## Etiology

Wilms tumor’s cause is not known accurately. Still, it is assumed that it occurs due to the alteration of genetics responsible for developing the genitourinary tract with normal embryological characteristics. About 1% of Wilms tumor patients have a relative with the disease who is conventionally not a parent (1).

Wilms tumor is assumed to progress from persistent nephrogenic rests or metanephric tissues. These may occur in the kidneys of infants (1%) but generally regress during childhood. In almost every case of bilateral tumors, these abnormal metanephric cells are found, but only 35% are observed in unilateral tumors (9). Hemihypertrophy, aniridia, and other urological disorders like horseshoe kidney, cryptorchidism, and hypospadias are linked with the malignancy. However, it is unclear if they play any significant role in the actual process of carcinogenesis. The bilateral disease depicts only about 5% of all patients with Wilms tumor and is more frequently seen in young females.

Wilms tumor is linked with various syndromes, including WAGR syndrome. WAGR syndrome refers to the presence of Wilms tumor, aniridia, genitourinary anomalies, and mental retardation. The chances of developing Wilms tumor in children with WAGR syndrome is 50%. A particular chromosomal abnormality in the WT1 gene relates to renal and gonadal development in children with this syndrome.

The Denys-Drash syndrome, or just Drash syndrome, is another syndrome associated with Wilms tumor. This involves male pseudo-hermaphroditism and advanced renal failure starting in infancy. This renal disease starts with basic proteinuria in new-born and infants, which in time advances to nephrotic syndrome and, due course, complete failure of the renal system. 90% of affected patients will eventually develop Wilms tumor (9).

## Histology

Histopathologically, Wilms tumor appears to be indistinguishable between adults and children. The genetic basis of Wilms tumor is complex. There is an association between the occurrence of Wilms tumor and disorders of chromosome 11 genes, such as WT1 and WT2 (5).

In 10% of tumors, the mutation of the WT1 gene, also denoted as 11p13, is seen. It also shows mutations 11p, 7p,16q, and 1p. Histologically, blastemal, epithelial, and mesenchymal cells are the hallmark characteristics observed in “favorable” Wilms tumors. In Wilms tumor, the prognosis of the blastemal dominant type of cancer is low. It is also more severe than other types of cancers. Intermediate-grade tumors feature epithelial and mesenchymal cells (5).

A high degree of anaplasia is seen in Wilms tumor with “unfavorable “histology. Predicting this type of tumor is arduous and has decreased survival rates. Anaplastic cells are observed with undifferentiated nuclei showcasing a high degree of variation in cellular size and shape, thrice the size of adjacent cells and depicting atypical mitotic cells (9).

WT1 expression was detected in the blastemal site and mature tissue but not in mesenchymal and mature tissue (6). The SIOP histological classification reflects changes resulting from chemotherapy, including “degenerative” changes. According to NWTSG, Wilms tumor is classified based on anaplasia. The revised SIOP classification of Wilms tumors is divided into three groups: low-risk, intermediate-risk, and high-risk (7). Histologically, Wilms tumor is classified into three risk groups, according to the SIOP 2001 protocols (8) (Table 1).

**Table 1:** Histological classification of Wilms tumor according to the risk groups - SIOP 2001 protocols.

Low-risk tumor (LR)	Intermediate risk tumor (IR)	High-risk tumor (HR)
Mesoblastic nephroma Completely necrotic Nephroblastoma Cystic partially differentiated Nephroblastoma	Epithelial type Stromal type Regressive type Mixed type Focal anaplasia	Blastema type Diffuse anaplasia Clear cell sarcoma of the kidney Rhabdoid tumor of the kidney

## Staging of Wilms Tumor

Based on the extent of tumor spread, the staging of Wilms’s tumor was classified from stage 1 to stage 5. Two different staging systems were developed by the National Wilms’ Tumor Study Group (NWTSG) and the International Society of Pediatric Oncology (SIOP). The survival rate is similar, over 90% for the patients treated with these two guidelines (4).

### Siop Protocol

#### Stage 1

In this stage, the tumor is restricted to the kidney or encircled by a fibrous pseudo capsule. The cancer may invade the renal capsule but not affect the exterior layer. The tumor is expunged and may be expanding into the pelvis and plunging into the ureter but does not penetrate its wall. The involvement of the renal sinus vessels is not spotted. Involvement of vessels inside the kidney may be observed.

#### Stage 2

The tumor expands past the kidney or invades through its capsule or fibrous pseudo capsule into fat around the kidney but is entirely expunged. The cancer invades the renal sinus and penetrates the blood and lymphatic vessels perirenal parenchyma but is expunged. The cancer invades nearby organs or vena cava but is entirely removed.

#### Stage 3

Partial abscission of the tumor, which expands past excision margins. Involvement of any lymph nodes of the abdomen is seen. Tumor disrupts before or intra-operatively. The tumor has invaded through the peritoneal surface. Tumor thrombi present at the excision margins of vessels or ureters, transected or resected gradually by the surgeon. The tumor has been surgically removed and sent to biopsy (specifically wedge biopsy) before starting chemotherapy or surgery.

#### Stage 4

Metastasis of the tumor (Brain, lungs, bone, liver, etc.) via blood or lymph nodes to the exterior regions of the abdomen and pelvis.

#### Stage 5

The tumor spreads to both kidneys at diagnosis.

### NWTSG Protocol

#### Stage 1

The tumor is restricted to the renal regions and expunged. The tumor was not disrupted before or during excision. The renal sinus vessels are involved but not over 2mm. Above the margins of the abscission, no residual tumor is seen.

#### Stage 2

The tumor expands on the far side of the renal region but is resected. No leftover tumor is evident at or past the margins of abscission. If the thrombus is expunged completely with the tumor, the tumor thrombus seen in vessels exterior to the renal region is stage 2.

#### Stage 3

The leftover tumor is limited to the abdominal region. Peritoneal contamination by the tumor is diffused by lymph nodes present in the renal hilum or the periaortic chains. On the surfaces of the peritoneum, implants are seen. The tumor expands over the margins of surgery either microscopically or glossy tumor is not resectable due to the local invasion into important structures.

#### Stage 4

Metastasis of the tumor via blood or distal lymph nodes is observed.

#### Stage 5

Involvement of both kidneys at the time of diagnosis.

## Evaluation

There are no specific diagnostic tests for adult Wilms tumors to confirm the disease or determine the tumor’s progression. But certain routine investigations include (9)

- Comprehensive blood test
- Biochemistry profile
- Renal function test
- Urinalysis
- Coagulation studies
- Cytogenetics studies to look for 1p and 16q deletion.

Imaging studies used include

- Renal ultrasonography
- Chest x-ray
- Chest and abdominal CT (with sedation)
- Abdominal MRI

Before planning surgery to remove the tumor, imaging tests are important. A Chest X-Ray is performed to check if the tumor is metastasized to the lungs. Abdominal CT and MRI also play a vital role in diagnosing Wilms tumor, so either can be used.

## Treatment

Wilms tumor is treated based on two standard protocols. They include SIOP and NWTSG/COG. The treatment commences with surgical abscission of the tumor followed by chemotherapy, according to NWTSG/COG studies. Complications involved with the surgery, including bowel obstruction, severe internal bleeding, infection from the wound, severe vascular injuries, and injuries to other visceral organs, were seen in NWTS – 4 study (4). In contrast, SIOP suggests commencing chemotherapy followed by surgery after 4 weeks. Only in specific conditions like developmental abnormalities in the adjacent kidney and genetic diseases in which the risk of development of renal tumor is high and in patients with only one kidney partial kidney abscission is allowed. This is following SIOP protocol (3). Another treatment option includes radical nephrectomy. In this procedure, the adrenal gland and lymph nodes are removed in addition to the removal of the kidney. Radical nephrectomy is the preferred treatment for one–sided renal tumors (10).

### Chemotherapy

The most promising chemotherapeutic agents to treat nephroblastoma are dactinomycin (DAM), carboplatin, doxorubicin (DOX), vincristine (VCR), ifosfamide (IF), cyclophosphamide (CPM), etoposide. These drugs are given as monotherapy or in combination. SIOP protocol suggests giving chemotherapy before the surgery to decrease the risk of tumor disruption at the time of surgery and hence decrease the recurrence of regional and distant tumors (10). NWTS recommends polychemotherapy (DAM, VCR, DOX) for fifteen weeks with additional treatment in stage 3 of the tumor. Less potent drugs (VCR and DAM) are preferred in stages 1 and 2 of cancer. Using the combination of three DAM, VCR, and DOX instead of the two–drug therapy consisting of DAM and VCR showed no superior improvements in stage 2 (11). Few changes were made in the pediatric regimen and used for the treatment of adults. But the rate of toxicity is higher in adults when compared to children. The therapeutic regimen for nephroblastoma in adults was assigned according to NWTS and SIOP guidelines (Table 2).

**Table 2:** Treatment protocols for Wilms tumor from NWTSG and SIOP studies.

NWTS – 5			
**Stage**	**Chemotherapy**		**Radiotherapy**
1 – FH^a^/UH^b^	DAM^c^/VCR^d^ x 18 weeks		—
2 – FH^a^	DAM^c^/VCR^d^ x 18 weeks		—
3-4 – FH^a^	DAM^c^/VCR^d^/DOX^e^ x 24 weeks		10.8 Gy^g^
2-4 – UH^b^	DAM^c^/VCR^d^/DOX^e^/CPM^f^/ Etoposide x 24 weeks		12 Gy^g^ lung (if the lung metastasis) 10.8 Gy^g^ flank (if local stage III)
**SIOP – 01**			
**Stage**	**Chemotherapy**		**Radiotherapy**
	**Preoperative**	**Postoperative**	
1	DAM^c^/VCR^d^ × 4 weeks	DAM^c^/VCR^d^ × 4 weeks	No
2	DAM^c^/VCR^d^ × 4 weeks	DAM^c^/VCR^d^/DOX^e^ × 27 weeks	Node negative: 15 Gy^g^
3	DAM^c^/VCR^d^ × 4 weeks	DAM^c^/VCR^d^/DOX^e^ × 27 weeks	15 Gy^g^
4	DAM^c^/VCR^d^/DOX^e^ × 6 weeks	CR^h^ after 9 weeks - DAM^c^/VCR^d^/DOX^e^ × 27 weeks. No CR^h^ after 9 weeks – ICED × 34 weeks	No need for RT^j^ if lung lesions disappear by week 9; otherwise, 12 Gy^g^

a FH – Favourable histology; ^b^UH – Unfavourable histology; ^c^DAM – Dactinomycin; ^d^VCR – Vincristine; ^e^DOX – Doxorubicin; ^f^CPM – Cyclophosphamide; ^g^Gy – Gray; ^h^CR – Complete response after treatment; ^i^ICED – Ifosfamide, Carboplatin, Etoposide, Doxorubicin; ^j^RT – Radiotherapy.

### Radiotherapy

Adult Wilms tumor is responsive to radiotherapy. Currently, only radiation therapy is used as a treatment for severe cases of Wilms tumors (stages 3, 4, and 5) and for some initial stages of tumors with unfavorable histology. According to NWTS and SIOP, the recommended dose of radiation is 10 and 15 Gy (Gray), respectively. In various SIOP trials such as SIOP1, SIOP2, and SIOP5, the benefits of preoperative radiation therapy in the avoidance of tumor disruption and in enhancing the distribution of stage was established. In the beginning, radiation therapy followed by chemotherapy cause the loss of the tumor cell volume (cell shrinkage) (10).

## Future Perspectives

Individualized treatment for adult Wilms tumors is the need of the hour. Developments in the field of epidemiology can help identify populations at risk in advance. Delay or confusion that persists with the diagnosis of Wilms tumor can decrease the survival rate in adults. But with humongous growth in molecular biology and tumor genetics, which can be expected in the upcoming years, the scope of identification of appropriate genetic and protein biomarkers with enhanced specificity will increase by multiple folds. This helps in the grouping of Wilms tumor patients in a well-defined spectrum. In addition, advancements in imaging studies are accompanied by the involvement of artificial intelligence, which can potentiate the timely diagnosis of adult Wilms tumors. Future developments in clinical studies of various target-specific drug molecules can be anticipated in the hope of treating the tumor in the best way possible. Along with improvements in artificial intelligence, the construction and programming of more efficient robots can bring about remarkable outcomes in surgical oncology, thereby increasing patients’ survival.

## Conclusion

The incidence of Wilms tumor is sparsely seen in adults. The meagerness of information concerning the diagnosis of Wilms tumor in adults causes a significant setback in managing the disease. As of now, several molecular prognostic factors have been found in children. With further research in the adult population, better clinical outcomes can be achieved. More studies with higher-quality evidence for the treatment are imperative. An individualized model of care and management of nephroblastoma in the adult population is indispensable. In the future, diversified research in the oncology field can help create ample scope with correspondence to the appropriation of treatment guidelines, improving the survival rate in the adult population.
